# All Wales Injury Surveillance System revised: development of a population-based system to evaluate single-level and multilevel interventions

**DOI:** 10.1136/injuryprev-2015-041814

**Published:** 2015-12-09

**Authors:** Ronan A Lyons, Samantha Turner, Jane Lyons, Angharad Walters, Helen A Snooks, Judith Greenacre, Ciaran Humphreys, Sarah J Jones

**Affiliations:** 1 Farr Institute, Swansea University Medical School, Swansea, UK; 2 Public Health Wales NHS Trust, Cardiff, UK

**Keywords:** Interventions

## Abstract

**Background:**

Injury surveillance has been established since the 1990s, but is still largely based upon single-source data from sentinel sites. The growth of electronic health records and developments in privacy protecting linkage technologies provide an opportunity for more sophisticated surveillance systems.

**Objective:**

To describe the evolution of an injury surveillance system to support the evaluation of interventions, both simple and complex in terms of organisation.

**Methods:**

The paper describes the evolution of the system from one that relied upon data only from emergency departments to one that include multisource data and are now embedded in a total population privacy protecting data linkage system. Injury incidence estimates are compared by source and data linkage used to aid understanding of data quality issues. Examples of applications, challenges and solutions are described.

**Results:**

The age profile and estimated incidence of injuries recorded in general practice, emergency departments and hospital admissions differ considerably. Data linkage has enabled the evaluation of complex interventions and measurement of longer-term impact of a wide range of exposures.

**Conclusions:**

Embedding injury surveillance within privacy protecting data linkage environment can transform the utility of a traditional single-source surveillance system to a multisource system. It also facilitates greater involvement in the evaluation of simple and complex healthcare and non-healthcare interventions and contributes to the growing evidence basis underlying the science of injury prevention and control.

## Introduction

In this paper, we describe the development of a population-based injury surveillance system to observe injury rates and patterns in order to develop and evaluate targeted interventions to gain maximum health impact.

Until fairly recently, injury prevention has been a neglected issue. The first world conference on injury prevention and control was not held until 1989 in Sweden and stimulated much action, including the need for better information on the scale, distribution and consequences of the problem, namely surveillance, which is derived from the French word ‘surveiller’ meaning ‘to watch over’. Injury surveillance has been promoted by a number of international organisations for several decades aided by a seminal guideline produced by the WHO and US Centers for Disease Control and Prevention in 2000.^1^

In most settings, surveillance is implemented passively as a policy tool to measure the scale of the problem and provide information on underlying causes to inform the development of generalised preventive approaches, rather than used proactively to support prevention at different levels. A number of countries and jurisdictions set up sentinel surveillance systems to quantify national-level or state-level incidence, but by their very nature these are rarely strongly linked to the targeting and evaluation of preventive and treatment efforts, much of which needs to be at a more local level. There are well-developed systems in operation in several settings, particularly in Australia, for example, Victoria Injury Surveillance Unit and the Queensland Injury Surveillance Unit, and in the USA, for example, Centres for Disease Control's Web-based Injury Statistics Query and Reporting System and the Florida Injury Surveillance Data System that now use multiple data sources.^2–5^

## Development of the All Wales Injury Surveillance System

In Wales, a fledgling emergency department (ED) injury surveillance system was developed in the West Glamorgan municipality (population 250 000) in 1993 and brought together data from the three EDs. This led to the creation of the All Wales Injury Surveillance System (AWISS) in 1998.^6^ ^7^ AWISS has always been a low-cost system based on patient management data as to this day it is difficult to convince policymakers of the importance and cost effectiveness of injury surveillance or even injury prevention initiatives given other pressures. AWISS was designed and implemented by public health practitioners and clinicians largely from ED backgrounds, initially as a tool to support local prevention efforts and subsequently to evaluate changes in the design and implementation of care.

## Impact of changing information governance rules

Changing information governance practices and norms have had substantial impact on AWISS over the years, particularly the development and implementation of the Caldicott Guidelines on NHS data protection in 1997 and the 1998 Data Protection Act. These perfectly reasonable developments were designed to protect privacy but led to considerable confusion and variation in interpretation between data controllers with a number declining to share even de-identified data. This adversely affected national coverage of AWISS from 2005 to 2009 until the Welsh government mandated a slimmed down Emergency Department Data Set (EDDS). However, this process involved mapping of codes to a standardised data set that does not quite fit and adversely affected data quality (see later section).

Using information from local treatment systems to target preventive interventions has also been somewhat controversial. In 1998, an individual interpretation of data protection legislation prevented us from passing on information on small areas (10–14 houses) that contained at least one home with high injury rates to local authority colleagues who wanted to initiate targeted home safety interventions.^8^ Despite no personal data being transferred, an intervention designed to protect poorer children at high risk was abandoned.

Thankfully, these information governance issues have largely been addressed by the 2nd Caldicott Report issued in 2013 that now includes a duty to share data, and the embedding of the surveillance system within the purposely designed, privacy protecting Secure Anonymised Information Linkage (SAIL) system.^9^ ^10^

## Development of multisource surveillance

Until 2008 AWISS was solely based on ED data from all major ED units. The development of the SAIL environment opened up opportunities to extending surveillance to other data sources.^9^ AWISS now uses a number of additional data sources including inpatient admission and outpatient data from the Patient Episode Database for Wales, data on the treatment of burns from the Welsh Centre for Burns and mortality data from the Office of National Statistics. Some 80% of general practices now also supply data.

**Figure 1:**
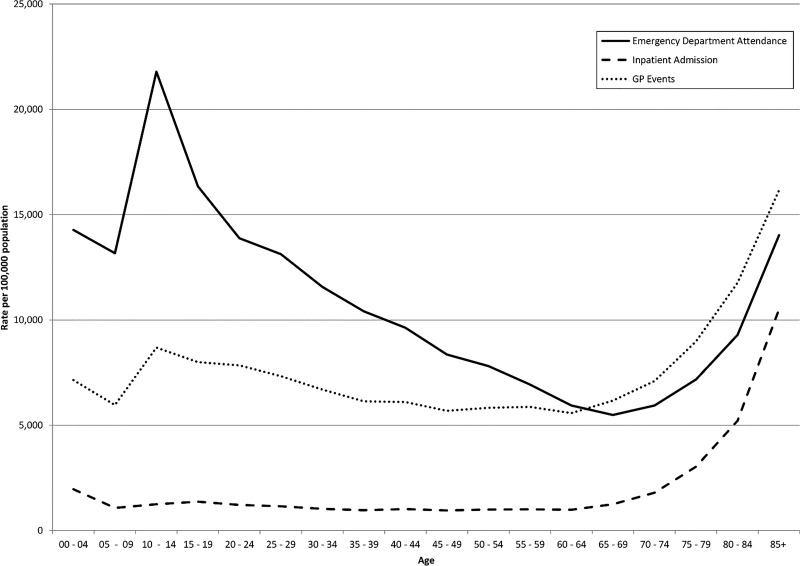
Rate of injury presentation by age group for Welsh residents per 100 000 population: general practice (GP) recorded events, emergency department attendances and inpatient admissions.

Figure 1 shows injury presentation rates for all injuries per 100 000 population in Wales for three data sets used by the injury surveillance system: ED attendances, general practice (GP) events and hospital admissions. Numbers and age-specific rates are shown in online supplementary table S1 with the codes used to define ED and hospital cases in appendices 1 and 2. The GP data uses Read codes that are only operational in the UK and New Zealand. There are 16 446 codes related to injury (available from the authors on request). Data patterns vary considerably by source and by age group.

**Figure 2:**
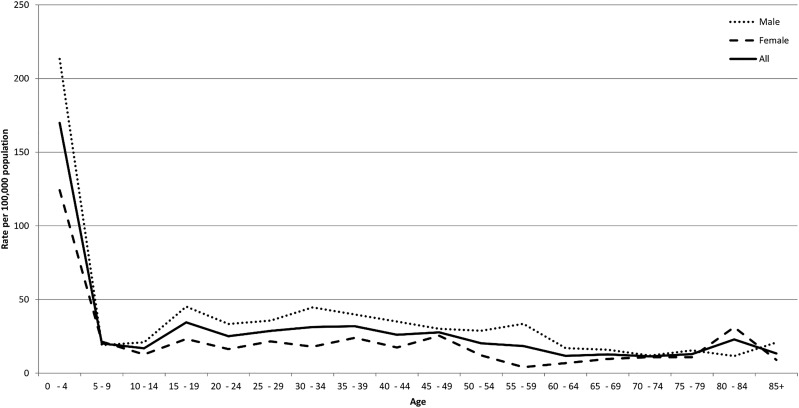
Rate of acute injury attendances to the Welsh Burns Centre by age and sex for Welsh residents per 100 000 population between April 2012 and March 2013.

Burns, for example, have a very different age and sex profile to all injuries and are dominated by scalds in the under 5s, as shown by data from the Welsh Burns Registry (figure 2).

Discussions are at an advanced stage to incorporate data from two relevant national audits that contain detailed data on injury diagnoses and severity that are missing from other data sets, the Trauma Audit and Research Network and the Intensive Care National Audit and Research Centre. There are also plans to incorporate ambulance service data when a new digital pen electronic patient clinical record data collection system becomes operational from September 2015.

## Data quality

One of the fundamental issues with systems such as AWISS that use routinely collected ED data without providing additional financial support to hospitals is variable data quality, with a considerable amount of missing data. Data quality is also adversely affected by mapping to a mandated national minimum data set designed more for performance management of waiting times than supporting injury prevention. However, data quality is also an issue in better planned and funded systems. For example, a study of home injuries that used data from the US NEISS system revealed a high proportion of missing location data.^11^

**Figure 3:**
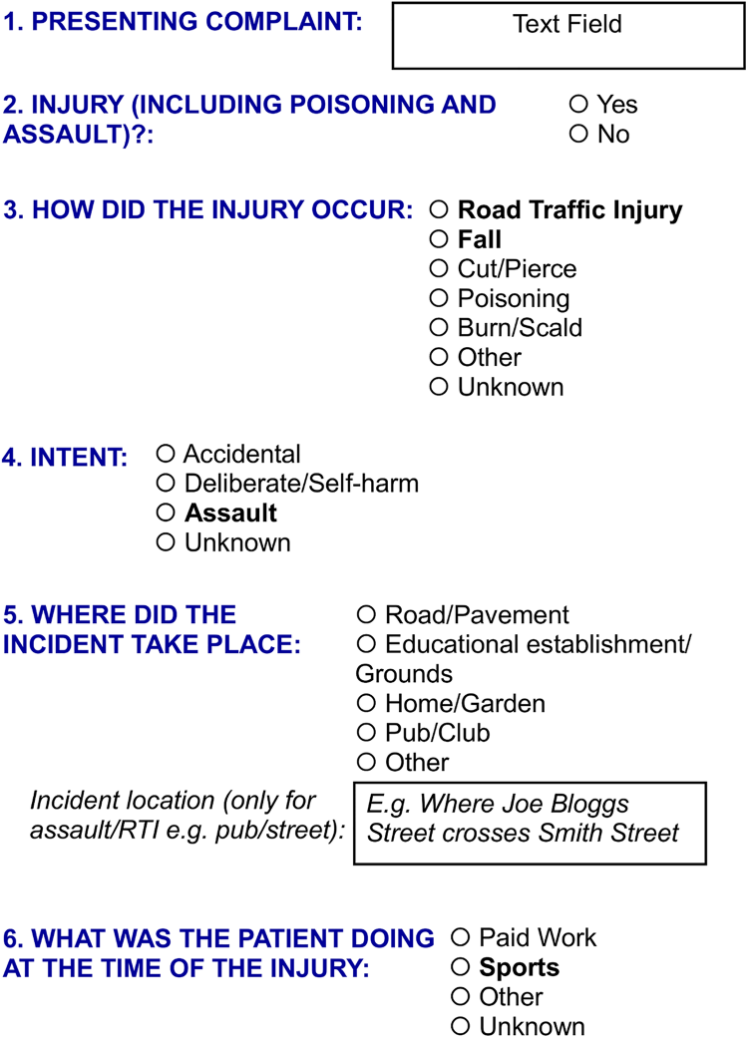
Single-screen Joint Action on Monitoring Injuries in Europe Minimum Data Set. Questions 3–6 only appear when ‘yes’ is selected in question 2.

Our philosophy has always been that imperfect data are still very valuable and to continuously work on improving data quality rather than give up at an early stage. Our approach has taken two forms, a push for standardisation at the source and the use of narrative data to fill gaps on location, intent, mechanism and activity. RAL and ST were instrumental in the development of the European Minimum Data Set (MDS) created by the Joint Action on Monitoring Injuries in Europe (JAMIE) project.^12^ ^13^ The JAMIE MDS is a single-screen system designed to capture data on injury aetiology in a way that eases data collection in busy EDs and improves data quality (figure 3). It is now being rolled out across Europe. The MDS contents were agreed following detailed discussions and consultations with ED clerks and clinicians, the European Injury Data Base national administrators and external experts from the USA and Australia. Although the system does not provide detailed information on all permutations of intent, activity, mechanism and location, it provides high-level data to enable the enumeration of injuries, which occur in the home, home and leisure (combined), at work, at school and on the road. It also distinguishes injuries resulting from falls, sports, poisoning, burns/scalds, and those caused by incidents, self-harm or assaults.

While the MDS single data collection screen (figure 3) contains only 5 questions and 14 useful responses (eg, excluding ‘other’ and ‘unknown’ responses that are useful for quality assurance but are otherwise uninformative), the combination of variables can derive up to 120 combinations of injury determinants. Furthermore, as injury data requirements vary by region/country, the MDS is designed to allow additional data items to be added where necessary. In figure 3, the options in bold have been expanded in Wales with additional screens to support prevention activities relating to assaults, road traffic injuries, falls and sports injuries. The Welsh government has recently mandated the inclusion of the JAMIE MDS in the new ED computer system being rolled out across Wales in 2015/2016.

Data linkage also provides enhanced insight into the varying quality and implications of missing codes from the source data. Figure 1 is based upon 334 007 ED cases with an injury diagnosis occurring in Welsh residents in 2013. A further 171 984 cases had codes suggestive of an injury (attendance due to incidents, assault, self-harm, undetermined and intent withheld), but with missing or null diagnostic codes, indicating that poor data collection at source or poor mapping of local to national codes may be substantial issues. The individual linkage system enabled mapping ED attendances to hospital inpatient data, identifying 32 324 emergency admissions on the same or next day, of which 8509 had a primary injury diagnosis on discharge coding (26.3%), suggesting that the system currently underestimates injuries by an estimated 45 273 cases (13.6% of the total). There seems to be confusion about the completion of the intent field within EDDS, and it appears that in some hospitals attendances due to medical conditions are being mapped to ‘unintentional (incidental) injuries' due to systems that provide aetiology fields for all attendances and not just injuries. The implementation of the research data appliances in hospitals will support direct access to the source data held within ED systems, hence avoiding third-party mapping that induces errors and should improve the quality of the system. It will also provide access to more detailed diagnosis codes because the new ED systems are designed to support data capture using SNOMED CT terms.

## Applications

AWISS has been used for many local initiatives, particularly identifying areas with high injury rates to make the case for preventive interventions, such as the provision of home safety equipment in low-income families. Many of these initiatives were too small and hence statistically underpowered to evaluate effectiveness using surveillance data alone. Many of the early scientific outputs of the systems were based around quantification of the scale of injury using ED data and comparisons with other jurisdictions, with a particular focus on inequalities.^14–18^ These descriptive epidemiological studies naturally led to a desire to move to interventional activity. The surveillance data were used to plan and evaluate a number of substantial interventions including the Wales Vitamin D Fracture Prevention Study, one of the largest fracture prevention trials that was subsequently incorporated into international meta-analyses, and the Advocacy for Pedestrian Safety Study, the first large-scale randomised trial of political advocacy in injury prevention.^19–21^

The development of the privacy protecting SAIL data linkage facility has been key to extending opportunities to engage in high-quality evaluation of simple and complex interventions.^9^ SAIL is one of only six high-quality data linkage systems worldwide recommended by a recent review of the field by the Council of Canadian Academies.^22^ It is also currently unique among the population-based data linkage systems in that it is capable of anonymising and linking data at multiple levels. All individuals in the population are assigned unique non-identifying ‘Anonymised Linkage Fields’ that are common across data sets. In addition, there are anonymised linkage fields for every residence, maternal-child links and encrypted organisational codes for schools, healthcare facilities and small area geographies. Hence, it is possible to embed individuals within multilevel hierarchies and evaluate simple or complex interventions operating at one or several levels. This is particularly important as there is growing support for the notion that some injuries may be better prevented by not primarily trying to prevent injury per se but by making environments and systems more salutogenic, resulting in fundamental changes to exposures at different levels.^23^ SAIL operates a complex split file approach that involves separation of identities from the rest of the data, separation of data flows, application of multiorganisational encryption and then reassembly of data to enable the system to operate while protecting privacy.^9^

The SAIL system has enabled us to begin to evaluate a number of complex interventions, trials and natural experiments that are likely to impact on different health, illness, injury and social outcomes. These include interventions to improve housing, evaluation of changes in exposure to alcohol outlet density, and simple or clustered health service trials, such as the Support and Assessment for Fall Emergency Referrals 1 (SAFER 1) and SAFER 2 randomised trials, which investigated the clinical and cost effectiveness of interventions for emergency ambulance paramedics to assess and refer older people following a fall to appropriate community-based care.^24–27^ SAFER 1 results demonstrated the higher follow-up rates achievable using linked routine data outcomes in a trial^26^ Primary and secondary outcomes from routine sources were retrieved for 69% (779/1123) of patients attended by emergency ambulances who met the inclusion criteria, 20% dissented from follow-up and 1% could not be matched; this compares with a response rate of 39% for self-reported outcomes retrieved by postal questionnaire.

Better information is also required to help understand the complexity of causal pathways and inter-relationships between exposures, interventions and outcomes in order to design more effective interventions. In particular, exposure data are needed to determine whether policies, such as addressing pedestrian safety, achieve their objectives (eg, fewer injuries) through increased safety or a reduction in an otherwise salutogenic exposure, walking. Exposure data that can be linked to interventions and outcomes are particularly scarce. As part of the European ‘Tools to Address Childhood Trauma, Injuries and Children's Safety’ project, we designed a web-based tool, ‘The School Travel and Child Safety Survey’ (STCSS), to assess a range of childhood exposure data.^28^ ^29^ STCSS is an online survey designed to be undertaken by children aged 10–13 years in school. It contains 20 questions covering travel to school, road, home, play and water safety, bullying and access to alcohol and cigarettes. The survey was initially piloted in schools across Europe to assess feasibility and reliability, including several schools in Neath Port Talbot (NPT) Council, a municipality in Wales.^30^ STCSS is now being implemented across all NPT schools, replacing a paper-based survey, and improving the quality, breadth and usability of data to support local active travel and road safety interventions. The web-based survey is designed to capture identities in a way that will support subsequent anonymisation and linkage to data in SAIL to facilitate medium-term and long-term evaluations of the effectiveness of active travel and child safety interventions on a range of health and social outcomes.^31^

## Future direction: seizing parallel opportunities

Involvement in the Farr Institute of Health Informatics Research, a multifunder research initiative designed to enhance research collaboration between academia, health services and industry, has brought additional resources including a wider range of expertise.^32^ Linkage of anonymised data allows sophisticated analysis of data from across disparate sources at an individual or residential level, with a further facility to tag participants recruited to trials and to link in questionnaire responses while still protecting individual identities. The Farr Institute investment allows us to work with Swansea Trials Unit to develop and implement innovative methodologies for data linkage within experimental research.

This investment is also stimulating research into exploiting the narrative contained in ED systems that often contains very rich information.^33^ We have developed a collaboration with Clinithink, a commercial organisation, to test the utility of developing natural language processing solutions to automatically provide SNOMEDCT, ICD10 codes (including Chapter 20) and bespoke codes on aetiology from ED narrative.^34^ We plan to extend this work to radiology reports and, if successful, to embed tools such as automated fracture classifications from the national radiology reporting software within AWISS, overcoming some of the limitations of ICD10 coding.

Additional investment through the Farr Institute to develop methodologies to increase research access to data trapped in isolated databases funded the development of research data appliances that will sit in multiple health care and non-healthcare organisations. These have the capability of characterising and linking data from multiple sources within an organisation and can export anonymised data to SAIL. While this technology has been developed as a generic research support tool, it will enable access to many isolated data sets held in health and social care organisations that otherwise would never be reached. These data sets often contain rich clinical material that will support more detailed evaluations of many health and non-healthcare interventions, including of course in the fields of injury prevention, treatment and rehabilitation.^35^ A stimulus to incorporating additional data sets has been the plans to use the SAIL system to evaluate the effectiveness of reconfigured trauma services, including the Emergency Medical and Retrieval Transport Service designed to hasten access to trained physicians and definitive care for the most seriously injured and ill patients.

## Conclusions

Persistence, serendipity and lateral thinking in seizing parallel opportunities have been important aspects of the survival, development and improvement of this injury surveillance system. The system is still far from perfect, but our philosophy is to strive for continual improvement. Our experience shows that embedding injury surveillance within a privacy protecting data linkage environment can transform the utility of a traditional single-source surveillance system to a multisourced system. This also facilitates greater involvement in the evaluation of simple and complex interventions in both healthcare and non-care settings and opens opportunities to leverage resources from parallel developments in other fields. It maximises the potential to engage in a wider range of research activities than the limited funds available through injury research programmes normally allows.^36^ Such a system could be adopted elsewhere and help contribute to the growing evidence basis underlying the science of injury prevention and control. We hope that this paper will serve as a useful resource and guideline for practitioners looking to advance and improve their current tools, while adhering to privacy concerns.

**What is already known on the subject**

- Injury surveillance has been in operation since the 1990s.
- Most existing surveillance systems are based on sentinel emergency department data; some use other data sets but do not link the data.

**What this study adds**

- Developments in privacy protecting data linkage can transform the potential for multisource injury surveillance opening up many opportunities.
- Population-based data linkage systems facilitate transition from observation to intervention and measurement of population impact.
- A long-term view of the value of surveillance is necessary.

## Supplementary Material

Web supplement
