# Supplementary material for: All Wales Injury Surveillance System revised: development of a population-based system to evaluate single-level and multilevel interventions
Source: Inj Prev. 2015 Dec 9;22(Suppl 1):i50–5. doi: 10.1136/injuryprev-2015-041814 (PMC4853534; doi:10.1136/injuryprev-2015-041814)
Supplement: Web supplement [file injuryprev-2015-041814-s1.pdf]

Supplementary Table1: Rates per 100,000 population for injury related GP events, ED attendances and inpatient admissions, in Wales.

Age	Injury Related GP Events[1] 01/01/2013 - 31/12/2013 (Rate per 100,000 Population)			Injury Related ED Attendance[2] 01/01/2014 - 31/12/2014 (counts)			Injury Related ED Attendances[3] 01/01/2014 - 31/12/2014 (Rate per 100,000 Population)			Injury Related Inpatient Admissions[4] 01/01/2014 - 31/12/2014 (Counts)			Injury Related Inpatient Admissions[5] 01/01/2014 - 31/12/2014 (Rate per 100,000 Population)		
	M	F	All	M	F	ALL	M	F	All	M	F	All	M	F	All
0 - 4	7768.39	6486.18	7147.55	14207	11163	25370	15598.55	12890.9	14278.88	1926	1555	3481	2114.65	1795.69	1959.20
5 - 9	6323.98	5580.82	5960.02	12664	10557	23221	14024.98	12271.59	13169.51	1149	750	1899	1272.48	871.81	1076.99
10 - 14	9836.92	7457.73	8684.22	20618	15360	35978	24275.61	19162.15	21792.83	1158	897	2055	1363.43	1119.04	1244.77
15 - 19	8698.06	7271.50	8000.94	18309	12489	30798	18856.79	13671.29	16343.06	1340	1238	2578	1380.09	1355.20	1368.02
20 - 24	8291.64	7368.39	7839.34	18156	12034	30190	16206.81	11401.12	13875.48	1668	982	2650	1488.93	930.36	1217.95
25 - 29	7828.49	6823.61	7332.89	15182	10038	25220	15564.26	10615.82	13128.51	1470	732	2202	1507.01	774.14	1146.27
30 - 34	6922.85	6445.62	6688.37	12431	8636	21067	13679.98	9469.091	11570.7	1189	684	1873	1308.46	749.98	1028.71
35 - 39	6059.84	6205.23	6131.00	9720	7729	17449	11669.65	9151.728	10401.97	997	624	1621	1196.98	738.86	966.34
40 - 44	5897.84	6319.49	6104.82	10170	8764	18934	10570.08	8734.216	9632.878	1201	802	2003	1248.25	799.27	1019.05
45 - 49	5354.61	6030.72	5686.54	9445	8763	18208	8860.225	7879.475	8359.464	1180	906	2086	1106.94	814.65	957.70
50 - 54	5221.20	6457.63	5829.81	8337	8517	16854	7875.347	7757.113	7815.152	1188	965	2153	1122.22	878.90	998.34
55 - 59	4848.38	6901.63	5875.71	6462	6893	13355	6852.307	7005.793	6930.678	1083	852	1935	1148.41	865.94	1004.18
60 - 64	4846.40	6306.33	5578.72	5296	5833	11129	5776.425	6085.55	5934.422	977	869	1846	1065.63	906.62	984.36
65 - 69	5116.81	7175.26	6164.04	4805	5788	10593	5094.576	5875.128	5493.354	1217	1193	2410	1290.34	1210.96	1249.79
70 - 74	5878.52	8221.93	7095.64	3757	4891	8648	5365.457	6470.176	5938.949	1244	1377	2621	1776.58	1821.60	1799.95
75 - 79	7642.64	10137.34	8981.93	3238	4983	8221	6089.099	8111.408	7173.084	1487	1988	3475	2796.32	3236.10	3032.05
80 - 84	9340.66	13600.40	11769.47	2713	4984	7697	7680.983	10491.97	9293.2	1617	2704	4321	4578.01	5692.27	5217.09

85+	14107.75	17211.13	16156.82	3042	8017	11059	11402.65	15361.77	14022.52	2545	5741	8286	9539.70	11000.61	10506.43
Total	6790.35	7402.04	7097.29	178552	155439	333991	11736.69	9896.029	10801.65	24636	24859	49495	1619.39	1582.65	1600.73

[1] Rates based on all injury related GP events (e.g. events may include letters and phone conversations in addition to visits). The list of injury related READ codes used in these figures can be requested from the author. Population estimates are based on the number of people registered with a SAIL GP practice on 31/07/2013.
[2] Counts of injury related Emergency Department attendances for Welsh residents (16 attendances were excluded as they lacked either sex or age demographics)
[3] Rates are based on injury related Emergency Department attendances for Welsh Residents. Population estimates based on ONS 2014 Mid-year estimates for Wales
[4] Counts of injury related inpatient admissions for Welsh Residents. The list of injury related ICD10 codes can be found in the appendix
[5] Rates are based on injury related inpatient admissions for Welsh Residents. Population estimates based on ONS 2014 Mid-year estimates for Wales

Appendix 1: Emergency Department Data Set codes used in the analysis to calculate counts and rates of Emergency Department attendances in Supplementary Table 1

**Injury codes**	**Injury Description**
01A	Laceration
01B	Contusion
01C	Abrasion
01D	Soft tissue inflammation
01Z	Wound, other or unspecified
	Head Injury
02A	Glasgow Coma Score 15
02B	Glasgow Coma Score <15
02C	Dental Injury
02Z	Head Injury, other or unspecified
	Fracture
03A	Open Fracture
03B	Closed Fracture
03C	Fracture Dislocation
03Z	Fracture, other or unspecified
	Joint Injury
04A	Sprain
04B	Dislocation
04C	Subluxation
04Z	Joint Injury, other or unspecified
	Amputation
05Z	Amputation, other or unspecified
	Soft Tissue Injury
06A	Muscle Injury
06B	Tendon Injury

06C	Nerve Injury
06D	Visceral Injury
06E	Vascular Injury
06Z	Soft Tissue Injury, other or unspecified
	Burns, Scalds and Thermal Conditions
07A	Electric
07B	Chemical
07C	Radiation
07D	Scald
07E	Sunburn
07F	Hyperthermia
07G	Hypothermia
07H	Frostbite
07Z	Burns, Scalds and Thermal Conditions, other or unspecified
	Foreign Body
08A	Ingested Foreign Body
08Z	Foreign Body, other or unspecified
	Puncture Wounds
09A	Needle Stick Injury
09B	Human Bite
09C	Animal Bite
09D	Insect Bite or Sting
09Z	Puncture Wounds, other or unspecified
	Poisoning or Overdose
10A	Alcohol
10B	Prescribed Drug
10C	Non-prescribed/purchased drug
10D	Illicit Drug
10Z	Poisoning or Overdose, other or unspecified
	Drowning
11A	Near Drowning

11Z	Drowning, other or unspecified
03Z	Wound closure
04Z	Removal foreign body
06Z	Manipulation

Appendix 2: Injury codes used to calculate counts and rate of injury related inpatient admissions

ICD10 injury codes	Sub-chapter description
S00-S99	Injuries to anatomical area
T00-T73, T75, T78	Injuries involving multiple body regions, Injuries to unspecified part of trunk, limb or body region, Effects of foreign body entering through natural orifice, Burns and corrosions, Frostbite, Poisoning by drugs, medicaments and biological substances, Toxic effects of substances chiefly nonmedicinal as to source, Unspecified effect of radiation, Effects of heat and light , Hypothermia , Other effects of reduced temperature, Effects of air pressure and water pressure, Asphyxiation, Effects of other deprivation, Effects of other external causes, Adverse effects, not elsewhere classified
V01-V99	Transport Accidents
W00- X59	Other external causes of accidental injury
X60-X84	Intentional self-harm
X85- Y09	Assault
Y10 - Y34	Event of undetermined intent
F10.0 - F19.0	Mental and behavioural disorders due to psychoactive substance use. *Only acute intoxication were included
